# Investigating the role of vitamin D in asthma

**DOI:** 10.7554/eLife.97031

**Published:** 2024-04-03

**Authors:** Siddhant Sharma, Mayank Garg

**Affiliations:** 1 Department of Biology, Ashoka University Sonipat India; 2 Simons Ashoka Fellowship Program, Trivedi School of Biosciences, Ashoka University Sonipat India; 3 Koita Centre for Digital Health, Trivedi School of Biosciences, Ashoka University Sonipat India

**Keywords:** Asthma, vitamin D receptor, inflammation, Th2 immune response, Human, Mouse

## Abstract

Results in mice suggest that vitamin D reduces the symptoms of asthma by controlling an immune response that leads to inflammation of the airways.

**Related research article** Kiliç A, Halu A, Marzio MD, Maiorino E, Duvall MG, Bruggemann T, Rojas Quintero JJ, Chase R, Mirzakhani H, Sungur AÖ, Koepke J, Nakano T, Peh HY, Krishnamoorthy N, Abdulnour RE, Georgopoulos K, Litonjua AA, Demay MB, Renz H, Levy BD, Weiss ST. 2023. Vitamin D constrains inflammation by modulating the expression of key genes on Chr17q12-21.1. *eLife* **13**:RP89270. doi: 10.7554/eLife.89270.

Vitamin D deficiency has burgeoned into a major public health concern, exacerbated by dietary habits and pollution among other factors (Cui et al., 2023). While vitamin D is well known to be important for maintaining healthy bones, it has also been linked with various immune disorders, including asthma.

Supplements of vitamin D are being increasingly used to treat immune-related conditions. However, it remains unclear precisely how vitamin D is able to improve the outcome of these disorders (Scragg, 2018). Now, in eLife, Scott Weiss, Ardu Halu and co-workers – including Ayşe Kiliç as joint first author with Halu – report how vitamin D regulates an immune response that is a major contributor to asthma (Kiliç et al., 2023).

First, the team – who are based at Brigham and Women’s Hospital and Harvard Medical School, and institutes in Germany, Japan, Russia, and the United States – revisited the findings of a clinical trial called the Vitamin D Antenatal Asthma Reduction Trial (VDAART). In the trial, pregnant women who had a history of Asthma or allergies (or whose partner, the other biological parent, had a similar history), were given low or high doses of vitamin D during pregnancy. Analyses of the data found that higher vitamin D supplementation did not significantly reduce asthma in the offspring (Litonjua et al., 2016; Litonjua et al., 2020). However, a more nuanced reanalysis – which adjusted for baseline vitamin D levels to account for factors such as dietary intake – reported a reduced risk of asthma in the offspring of women who received a higher dose of vitamin D during pregnancy (Wolsk et al., 2017).

Kiliç et al. set out to find the genetic underpinnings of this protective effect, focusing their attention on chromosome 17, which contains regions strongly associated with asthma and other immune diseases (Bansal et al., 2021). Across chromosome 17 are sites where the receptor for vitamin D (known as VDR) can bind. Bioinformatic analysis revealed that some of these VDR binding sites overlapped with genetic variants associated with diseases triggered by the immune response mediated by T helper type 2 cells. These immune cells (known as Th2 for short) are a subset of white blood cells which respond proactively to invading pathogens like helminths, as well as recurrent exposures. Th2 cells act by producing inflammatory mediators as well as by modulating the activity of other cells in the immune system. They are also activated by allergens, such as mites and pollens, which can lead to allergic inflammation and disorders like asthma.

A detailed exploration of these overlapping regions suggested that VDR can trigger a cascade that regulates genes involved in the Th2 immune response. This genetic regulation could either promote or repress the Th2 response, depending on other genetic variants present in the proximity.

With a hypothesis established, the researchers tested their findings in mice which had been exposed to extracts from house dust mites to mimic the asthma phenotype (Figure 1A). They found that mice deficient in vitamin D or lacking VDRs displayed a more severe phenotype. Further experiments revealed that house dust mite exposure also caused the Th2 cells to express higher levels of VDR. When these Th2 cells were exposed to calcitriol (the active form of vitamin D), the VDRs bound to the calcitriol and migrated from the cytosol into the nucleus (Figure 1B).

**Figure 1.**
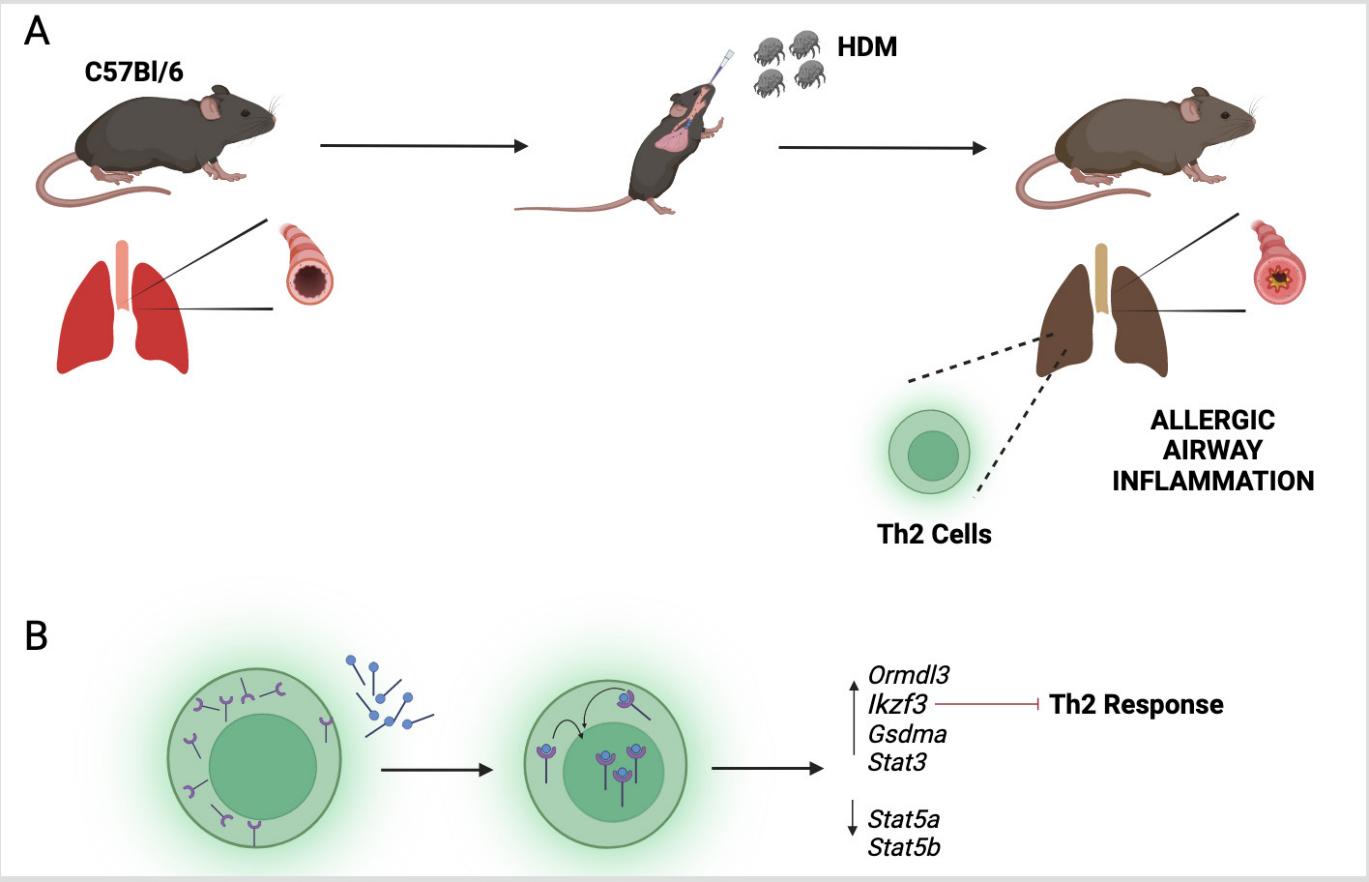
The vitamin D receptor and its role in inflammation of the airways. (**A**) To mimic the symptoms of asthma, Kiliç et al. treated a lab-grown strain of mice (known as C57Bl/6) with extracts from house dust mites (HDM). This activated a group of immune cells called T helper type 2 (Th2; green) in the lungs of the mice, leading to inflammation of their airways. (**B**) Kiliç et al. found that exposure to dust mites also caused the Th2 cells to produce more vitamin D receptors (purple). When the cells were treated with calcitriol, the active form of vitamin D (blue circles with black lines), the receptors migrated from the cytosol to the nucleus. Once there, the receptor can regulate the expression of genes involved in the Th2 response, including the gene *Ikzf3* which suppresses the inflammatory response triggered by Th2 cells.

These findings suggest that VDR acts like a lock waiting for its key. Access to the key (vitamin D), and migration of the VDR into the nucleus, possibly unlocks the transcriptional regulation required to modulate the immune pathways. Furthermore, VDR expression was contingent upon baseline vitamin D levels, suggesting that this vitamin has both a preventive and therapeutic potential. Kiliç et al. also found that one of the genes that VDR regulates (called *Ikzf3*) is a major factor in suppressing the Th2 immune response (Figure 1B). This effect is likely mediated through the STAT signaling axis, which is a critical pathway regulating inflammation (Hu et al., 2021).

The study by Kiliç, Halu, Weiss and colleagues sheds light on how vitamin D offers protective benefits against asthma. However, several pathophysiological pathways can lead to the characteristic airway inflammation associated with asthma (Moore and Bleecker, 2014). Subgroup analysis during clinical trials, along with targeted exploration of relevant biomarkers, could help identify who would benefit most from vitamin D supplements. The latest findings also underscore the importance of vitamin D in a wider sense, as the Th2 response is associated with several other chronic inflammatory diseases.

That being said, it is essential to be mindful of the broader biological role of vitamin D, as it influences both the innate and the adaptive immune system via a number of different cell types (Colotta et al., 2017). This complexity may be why scientific literature on vitamin D is marred by contradictory findings: for instance, a previous study has even shown loss of VDR to be protective against asthma (Wittke et al., 2004). However, this complexity should not deter researchers from trying to develop a deeper mechanistic understanding of vitamin D effects. Such an understanding could, in the future, enable personalized treatment strategies for individuals with immune disorders such as asthma.

## References

[bib1] Bansal M, Garg M, Agrawal A (2021). Advances in asthma genetics. Advances in Genetics.

[bib2] Colotta F, Jansson B, Bonelli F (2017). Modulation of inflammatory and immune responses by vitamin D. Journal of Autoimmunity.

[bib3] Cui A, Zhang T, Xiao P, Fan Z, Wang H, Zhuang Y (2023). Global and regional prevalence of vitamin D deficiency in population-based studies from 2000 to 2022: A pooled analysis of 7.9 million participants. Frontiers in Nutrition.

[bib4] Hu X, Li J, Fu M, Zhao X, Wang W (2021). The JAK/STAT signaling pathway: from bench to clinic. Signal Transduction and Targeted Therapy.

[bib5] Kiliç A, Halu A, Marzio MD, Maiorino E, Duvall MG, Bruggemann T, Rojas Quintero JJ, Chase R, Mirzakhani H, Sungur AÖ, Koepke J, Nakano T, Peh HY, Krishnamoorthy N, Abdulnour RE, Georgopoulos K, Litonjua AA, Demay MB, Renz H, Levy BD, Weiss ST (2023). Vitamin D constrains inflammation by modulating the expression of key genes on Chr17q12-21.1. eLife.

[bib6] Litonjua AA, Carey VJ, Laranjo N, Harshfield BJ, McElrath TF, O’Connor GT, Sandel M, Iverson RE, Lee-Paritz A, Strunk RC, Bacharier LB, Macones GA, Zeiger RS, Schatz M, Hollis BW, Hornsby E, Hawrylowicz C, Wu AC, Weiss ST (2016). Effect of prenatal supplementation with vitamin D on asthma or recurrent wheezing in offspring by age 3 Years: The VDAART randomized clinical trial. JAMA.

[bib7] Litonjua AA, Carey VJ, Laranjo N, Stubbs BJ, Mirzakhani H, O’Connor GT, Sandel M, Beigelman A, Bacharier LB, Zeiger RS, Schatz M, Hollis BW, Weiss ST (2020). Six-year follow-up of a trial of antenatal vitamin D for asthma reduction. The New England Journal of Medicine.

[bib8] Moore WC, Bleecker ER (2014). Asthma heterogeneity and severity-why is comprehensive phenotyping important?. Lancet Respiratory Medicine.

[bib9] Scragg R (2018). Limitations of vitamin D supplementation trials: Why observational studies will continue to help determine the role of vitamin D in health. The Journal of Steroid Biochemistry and Molecular Biology.

[bib10] Wittke A, Weaver V, Mahon BD, August A, Cantorna MT (2004). Vitamin D receptor-deficient mice fail to develop experimental allergic asthma. Journal of Immunology.

[bib11] Wolsk HM, Harshfield BJ, Laranjo N, Carey VJ, O’Connor G, Sandel M, Strunk RC, Bacharier LB, Zeiger RS, Schatz M, Hollis BW, Weiss ST, Litonjua AA (2017). Vitamin D supplementation in pregnancy, prenatal 25(OH)D levels, race, and subsequent asthma or recurrent wheeze in offspring: Secondary analyses from the Vitamin D Antenatal Asthma Reduction Trial. The Journal of Allergy and Clinical Immunology.

